# Age-stratified analysis of the BMI-kidney stone relationship: findings from a national cross-sectional study

**DOI:** 10.3389/fmed.2025.1513799

**Published:** 2025-02-12

**Authors:** Liuliu Zhou, Wei Gu, Yufeng Jiang, Haimin Zhang

**Affiliations:** ^1^Tongji University School of Medicine, Shanghai, China; ^2^Department of Nursing, Chongming Branch, Shanghai Tenth People’s Hospital, Tongji University School of Medicine, Shanghai, China; ^3^Department of Urology, Chongming Branch, Shanghai Tenth People’s Hospital, Tongji University School of Medicine, Shanghai, China; ^4^Department of Urology, Shanghai Tenth People’s Hospital, Tongji University School of Medicine, Shanghai, China

**Keywords:** body mass index, kidney stones, age-stratified analysis, obesity, public health, NHANES

## Abstract

**Background:**

The association between body mass index (BMI) and kidney stone formation may vary across different age groups and follow nonlinear patterns.

**Methods:**

This study analyzed data from NHANES 2009–2018, including 14,880 participants aged ≥20 years, to evaluate the association between BMI and the risk of kidney stones. BMI was categorized as normal weight (<25.0 kg/m^2^), overweight (25.0–29.9 kg/m^2^), and obesity (≥30.0 kg/m^2^). Weighted logistic regression models were employed to adjust for multiple confounders, including sex, age, race/ethnicity, education level, smoking history, alcohol consumption, coronary heart disease, diabetes mellitus, hypertension, and physical activity. Interaction effects between BMI and key variables such as sex, race/ethnicity and other factors were also analyzed. Age-stratified analyses were performed for the groups aged 20–39 years, 40–59 years, and ≥ 60 years. A restricted cubic spline model was used to explore the non-linear relationship between BMI and the risk of kidney stones.

**Results:**

After adjusting for confounders, participants with a BMI ≥30 kg/m^2^ had a significantly higher risk of kidney stones compared to those with a BMI <25 kg/m^2^ (adjusted OR [aOR]: 1.86; 95% CI: 1.48–2.34; *p* < 0.001), with the association being most pronounced in the 40–59-year age group (aOR: 2.02; 95% CI: 1.36–3.02; *p* < 0.001). The interaction analysis did not reveal significant interactions between BMI and sex or other factors. Non-linear analysis indicated that the relationship between BMI and kidney stone risk differed across age groups. In the 40–59-year group, the risk of kidney stones peaked and then plateaued as BMI increased beyond a certain threshold. In the ≥60-year age group, risk initially increased with BMI but then slightly declined. This non-linear relationship suggests that the impact of BMI on kidney stone risk varies by age and should be considered in clinical strategies.

**Conclusion:**

High BMI is significantly associated with an increased risk of kidney stones, particularly among middle-aged and older adults. The relationship between BMI and kidney stones is non-linear, highlighting the need to develop age-specific BMI management strategies to reduce the occurrence of kidney stones.

## 1 Introduction

Kidney stones affect 1 to 20% of the global population and approximately 11% of adults in the United States, with nearly 50% of affected individuals experiencing recurrence within 5 years (1–3). In the United States, kidney stones account for an estimated 2 million emergency department visits and $10 billion in annual health care costs (4).

Obesity, which affected more than 42% of U.S. adults as of 2017–2018, is a growing public health concern and a well-established risk factor for kidney stone formation (5, 6). Obesity-related metabolic disturbances, including hypercalciuria, hyperuricosuria, and hypocitraturia, promote crystal aggregation and growth, while insulin resistance disrupts urinary acid–base homeostasis, compounding the risk of kidney stone development (7–10).

Body mass index (BMI), the most widely used metric for assessing obesity, has been extensively studied in relation to kidney stone formation. Previous analyses of NHANES data have identified both linear and nonlinear associations between BMI and kidney stone risk, but the potential moderating role of age remains insufficiently explored (11, 12). Physiological changes associated with aging—including declining renal function, hormonal fluctuations (e.g., menopause), and obesity-related comorbidities such as hypertension and diabetes—may modify the relationship between BMI and kidney stone formation, particularly in older adults (8, 13, 14). In contrast, dietary and metabolic disruptions play a more significant role in kidney stone formation among younger individuals, with stone composition varying across age groups (15, 16).

This study examines the linear and nonlinear relationships between BMI and kidney stone formation across age groups, utilizing NHANES data. By clarifying the role of age as a modifier, these findings aim to inform targeted prevention and treatment strategies for obesity-related kidney stones.

## 2 Materials and methods

### 2.1 Study design and data source

This cross-sectional study utilized data from the NHANES from 2009 to 2018. NHANES is a nationally representative survey of the non-institutionalized U.S. population, employing a complex, multistage probability sampling design. The survey combines interviews, physical examinations, and laboratory tests to assess the health and nutritional status of participants. Details of the survey design and methodology can be publicly achieved from website.^1^

### 2.2 Study population

**Figure 1:**
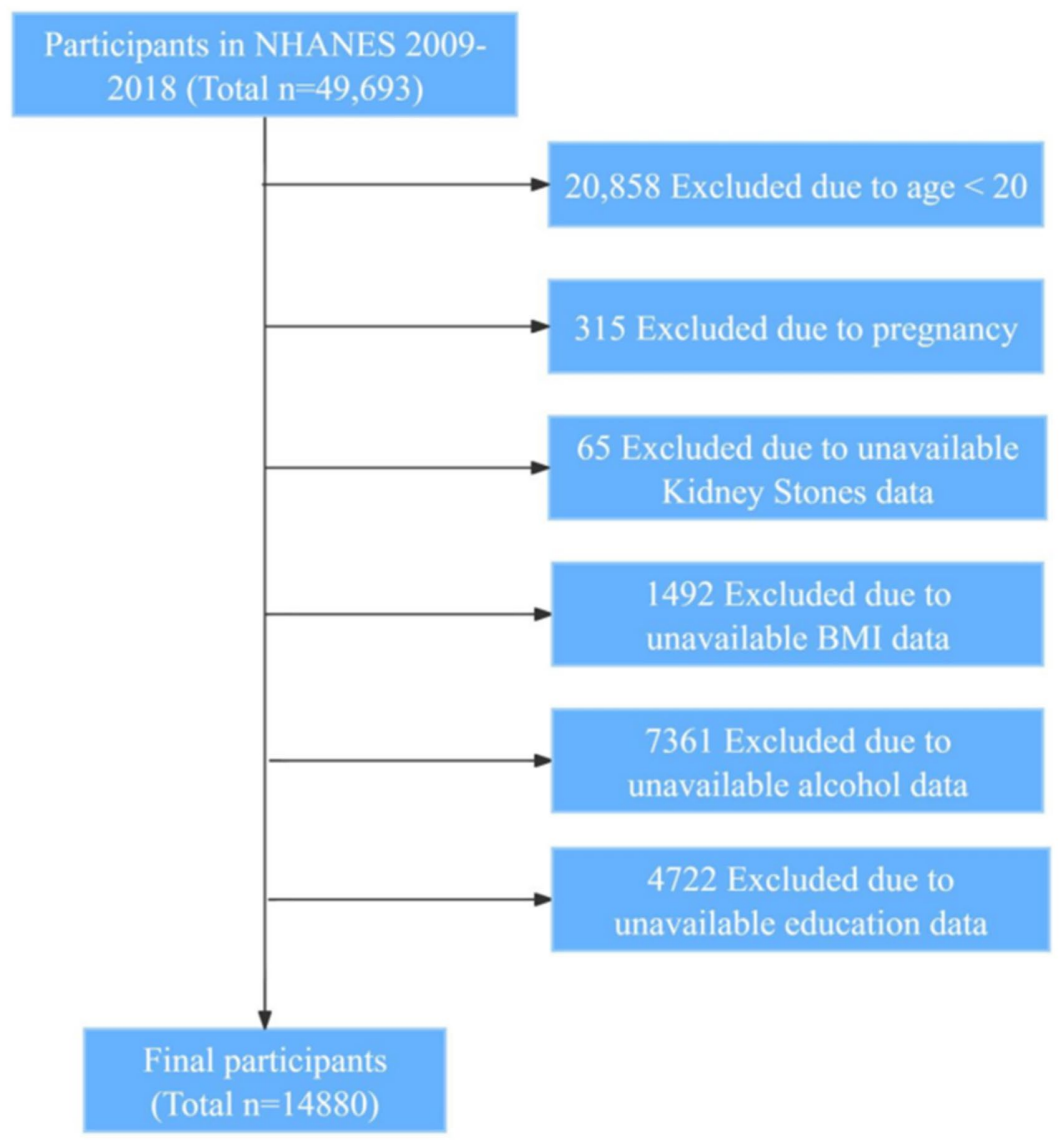
Flowchart of participant selection. NHANES, National Health and Nutrition Examination Survey; BMI, body mass index.

Participants aged 20 years and older were included in the analysis. Exclusion criteria were missing key data (BMI, kidney stone history) and pregnancy due to physiological changes affecting BMI. After excluding incomplete cases and ensuring less than 15% missing data for each variable, the final sample consisted of 14,880 participants. Missing covariate data were handled using multiple imputation techniques. Figure 1 illustrates the participant selection process.

### 2.3 Assessment of BMI

BMI was calculated using measured weight and height obtained during the NHANES physical examination. Weight was measured to the nearest 0.1 kg using a calibrated digital scale, and height was measured to the nearest 0.1 cm using a stadiometer. BMI was calculated as weight in kilograms divided by height in meters squared (kg/m^2^). Participants were categorized into three BMI groups: Normal weight: BMI < 25.0 kg/m^2^; Overweight: 25.0 ≤ BMI < 30.0 kg/m^2^; Obese: BMI ≥ 30.0 kg/m^2^.

### 2.4 Assessment of kidney stone history

Kidney stone history was determined based on self-reported data from the Kidney Conditions questionnaire. Participants were asked, “KIQ026—Ever had kidney stones?” Those who answered “Yes” were classified as having a history of kidney stones. Previous studies have validated the reliability of self-reported kidney stone history in epidemiological research (17).

### 2.5 Covariates

Potential confounders were selected based on clinical relevance and previous literature. Demographic variables included age, sex (male or female), race/ethnicity (Mexican American, Other Hispanic, Non-Hispanic White, Non-Hispanic Black, and Other Race—including Multi-Racial), education level (less than high school, high school graduate, above high school), and poverty income ratio (PIR). Lifestyle factors included smoking status (never, former, current), alcohol consumption (yes or no), and moderate/vigorous recreational activities (assessed through the “Physical Activity” section of the NHANES questionnaire, defined as moderate or vigorous activities lasting at least 10 consecutive minutes). Clinical variables included coronary heart disease, diabetes mellitus, and hypertension, all based on diagnoses recorded by physicians or other healthcare professionals in the questionnaire.

### 2.6 Statistical analysis

All statistical analyses were conducted using R version 4.2.3 (R Foundation for Statistical Computing, Vienna, Austria) and Python 3.8 (Python Software Foundation, United States) for data processing and visualization. The analyses utilized key libraries including *tidyverse* and *survey* in R for data manipulation and survey design analysis, and *pandas*, *statsmodels*, and *matplotlib* in Python for data processing, statistical modeling, and visualization. The analyses accounted for the complex survey design and sampling weights of the NHANES dataset to ensure nationally representative estimates. A two-sided *p*-value of less than 0.05 was considered statistically significant. Continuous variables were expressed as mean ± standard deviation (SD) and compared using *t*-tests. Categorical variables were presented as frequencies and percentages and compared using chi-square tests. The prevalence of kidney stones across different age and BMI categories was calculated using sampling weights. Weighted logistic regression models were used to examine the association between BMI categories and kidney stone prevalence. Three models were constructed: Model 1: Unadjusted model (univariate analysis). Model 2: Adjusted for sex, race/ethnicity, education level, and PIR. Model 3: Further adjusted for smoking status, alcohol consumption, coronary heart disease, DM, high blood pressure, and moderate/vigorous recreational activities.

#### 2.6.1 Age-stratified analysis

Participants were stratified into three age groups: 20–39 years, 40–59 years, and ≥ 60 years. Separate logistic regression analyses were conducted within each age group. Interaction terms between BMI categories and covariates were included in the logistic regression models to test for potential effect modification. Results were visualized using forest plots. Restricted cubic spline (RCS) models were used to assess potential non-linear associations between BMI and kidney stone risk. Knots were placed at the 5th, 50th, and 95th percentiles of the BMI distribution, following standard recommendations (18). Separate RCS models were constructed for each age group, adjusted for the same covariates as in Model 3. The fit of the logistic regression models and RCS models was compared using Akaike Information Criterion (AIC) and Bayesian Information Criterion (BIC). Lower AIC and BIC values indicate a better model fit.

### 2.7 Ethical considerations

NHANES protocols were approved by the National Center for Health Statistics Research Ethics Review Board, and all participants provided written informed consent. This study involved secondary analysis of publicly available, de-identified data and was exempt from additional ethical review.

## 3 Results

### 3.1 Baseline characteristics of the participants

In the NHANES 2009–2018 adult population, individuals with kidney stones were older and had higher BMI compared to those without kidney stones. Specifically, 49.6% of kidney stone patients had a BMI ≥30 kg/m^2^, compared to 40.5% of non-stone patients (*p* < 0.001). Table 1 provides an overview of the key demographic and clinical characteristics.

**Table 1:** Baseline characteristics of kidney stone and non-stone patients (NHANES 2009–2018).

Characteristic	None-stone *N* (%)	Stone *N* (%)	*p*-value
Total patients	13,397	1,483	–
Age (mean ± SD)	49.37 ± 18.05	55.78 ± 16.04	<0.001
BMI (mean ± SD)	29.5 ± 7.1	31.2 ± 7.3	<0.001
Sex			0.035
Male	6,797 (50.7)	709 (47.8)	
Female	6,600 (49.3)	774 (52.2)	
Race/Ethnicity			<0.001
Mexican American	2,426 (18.1)	233 (15.7)	
Other Hispanic	1,536 (11.5)	198 (13.4)	
Non-Hispanic White	5,047 (37.7)	748 (50.4)	
Non-Hispanic Black	3,209 (24.0)	207 (14.0)	
Other Race	1,179 (8.8)	97 (6.5)	
Education Level			0.338
Less than high school	4,190 (31.3)	455 (30.7)	
High school	3,943 (29.4)	417 (28.1)	
Above high school	5,264 (39.3)	611 (41.2)	
PIR			0.054
<1.0	3,344 (25.0)	334 (22.5)	
1.0 ≤ PIR < 3.0	6,900 (51.5)	768 (51.8)	
≥3.0	3,153 (23.5)	381 (25.7)	
Smoking			<0.001
Never	6,939 (51.8)	658 (44.4)	
Former	3,176 (23.7)	484 (32.6)	
Current	3,282 (24.5)	341 (23.0)	
Drinking			0.807
Yes	9,441 (70.5)	1,040 (70.1)	
No	3,956 (29.5)	443 (29.9)	
Coronary heart disease			<0.001
Yes	515 (3.8)	129 (8.7)	
No	12,882 (96.2)	1,354 (91.3)	
Diabetes mellitus			<0.001
Yes	1818 (13.6)	361 (24.3)	
No	11,579 (86.4)	1,122 (75.7)	
High blood pressure			<0.001
Yes	5,109 (38.1)	794 (53.5)	
No	8,288 (61.9)	689 (46.5)	
Moderate/Vigorous recreational activities			<0.001
Yes	5,761 (43.0)	504 (34.0)	
No	7,636 (57.0)	979 (66.0)	
BMI Category			<0.001
<25	3,635 (27.1)	251 (16.9)	
25 ≤ BMI<30	4,340 (32.4)	496 (33.4)	
≥30	5,422 (40.5)	736 (49.6)	

Continuous variables are presented as mean (SD) and were compared using *t*-tests. Categorical variables are presented as *N* (%) and were compared using chi-square tests. BMI, body mass index; PIR, poverty income ratio.

### 3.2 Baseline characteristics stratified by age groups

Table 2 presents the baseline characteristics stratified by age groups (20–39, 40–59, and ≥ 60 years). Significant differences were observed across age groups in BMI, sex, race/ethnicity, smoking status, alcohol consumption, and prevalence of coronary heart disease, high blood pressure, and diabetes mellitus (all *p* < 0.001). Notably, the proportion of individuals with a BMI ≥30 was highest in the 40–59 age group (45.9%) and slightly lower in the ≥60 age group (41.0%), compared to the 20–39 age group (37.0%).

**Table 2:** Baseline characteristics stratified by age groups (NHANES 2009–2018).

Characteristic	Age 20–39 *N* (%)	Age 40–59 *N* (%)	Age ≥ 60 *N* (%)	*p*-value
Total patients	4,580	4,861	5,439	–
Age (mean ± SD)	28.42 ± 5.68	48.47 ± 5.68	69.56 ± 7.10	<0.001
BMI (mean ± SD)	29.0 ± 7.7	30.5 ± 7.3	29.6 ± 6.5	<0.001
Sex				<0.001
Male	2,437 (53.2%)	2,376 (48.9%)	2,561 (47.1%)	
Female	2,143 (46.8%)	2,485 (51.1%)	2,878 (52.9%)	
Race/Ethnicity				<0.001
Mexican American	914 (20.0%)	934 (19.2%)	811 (14.9%)	
Other Hispanic	519 (11.3%)	572 (11.8%)	643 (11.8%)	
Non-Hispanic White	1,654 (36.1%)	1780 (36.6%)	2,361 (43.4%)	
Non-Hispanic Black	1,034 (22.6%)	1,162 (23.9%)	1,220 (22.4%)	
Other Race	459 (10.0%)	413 (8.5%)	404 (7.4%)	
Education Level				<0.001
Less than high school	1,065 (23.3%)	1,567 (32.2%)	2013 (37.0%)	
High school	1,374 (30.0%)	1,405 (28.9%)	1,581 (29.1%)	
Above high school	2,141 (46.7%)	1889 (38.9%)	1845 (33.9%)	
PIR				<0.001
<1.0	1,412 (30.8%)	1,185 (24.4%)	1,081 (19.9%)	
1.0 ≤ PIR < 3.0	2,284 (49.9%)	2,376 (48.9%)	3,008 (55.3%)	
≥3.0	884 (19.3%)	1,300 (26.7%)	1,350 (24.8%)	
Smoking				<0.001
Never	2,632 (57.5%)	2,392 (49.2%)	2,573 (47.3%)	
Former	576 (12.6%)	1,042 (21.4%)	2042 (37.5%)	
Current	1,372 (30.0%)	1,427 (29.4%)	824 (15.1%)	
Drinking				<0.001
Yes	1,085 (23.7%)	1,295 (26.6%)	2019 (37.1%)	
No	3,495 (76.3%)	3,566 (73.4%)	3,420 (62.9%)	
Coronary heart disease				<0.001
Yes	4,565 (99.7%)	4,772 (98.2%)	4,899 (90.1%)	
No	15 (0.3%)	89 (1.8%)	540 (9.9%)	
Diabetes mellitus				<0.001
Yes	4,464 (97.5%)	4,214 (86.7%)	4,023 (74.0%)	
No	116 (2.5%)	647 (13.3%)	1,416 (26.0%)	
High blood pressure				<0.001
Yes	602 (13.1%)	1776 (36.5%)	3,525 (64.8%)	
No	3,978 (86.9%)	3,085 (63.5%)	1914 (35.2%)	
Moderate/Vigorous recreational activities				<0.001
Yes	2086 (45.5%)	2,892 (59.5%)	3,637 (66.9%)	
No	2,494 (54.5%)	1969 (40.5%)	1802 (33.1%)	
BMI Category				<0.001
<25	1,560 (34.1%)	1,040 (21.4%)	1,286 (23.6%)	
25 ≤ BMI<30	1,324 (28.9%)	1,590 (32.7%)	1922 (35.3%)	
≥30	1,696 (37.0%)	2,231 (45.9%)	2,231 (41.0%)	

Continuous variables are presented as mean (SD) and were compared using *t*-tests. Categorical variables are presented as *N* (%) and were compared using chi-square tests. BMI, body mass index; PIR, poverty income ratio.

### 3.3 Distribution of weighted kidney stone prevalence across age and BMI categories

**Figure 2:**
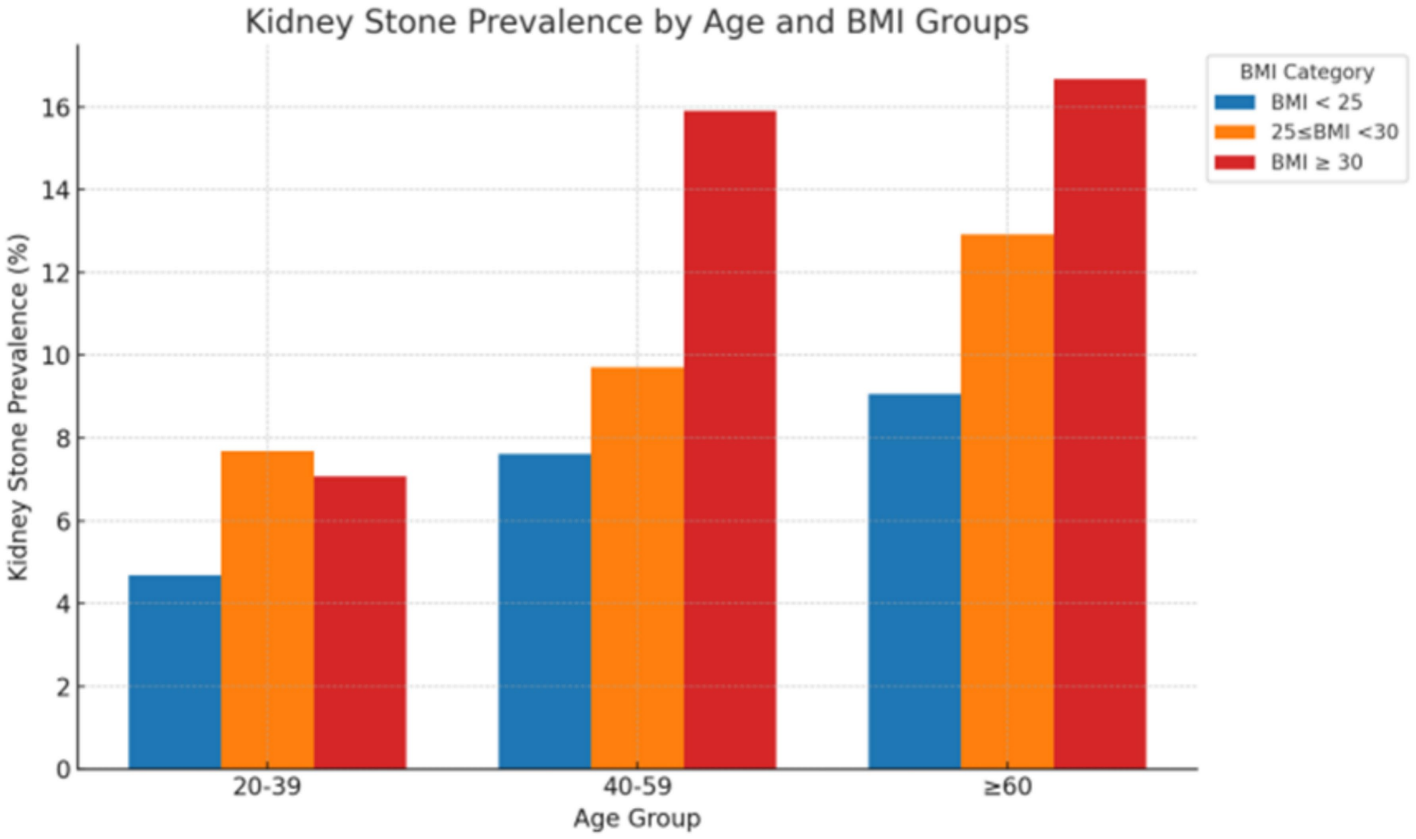
Prevalence of kidney stones by age group and BMI category.

Figure 2 illustrates the prevalence of kidney stones across different age groups and BMI categories, based on weighted-adjusted data. The prevalence of kidney stones increases with higher BMI in age ≥ 40 groups, particularly in individuals aged ≥60 years, where the prevalence in the BMI ≥ 30 kg/m^2^ group approaches 17%.

### 3.4 Logistic regression analysis of kidney stone risk by age and BMI

Weighted logistic regression analyses were conducted for the entire sample and stratified by age groups using the three models, as shown in Table 3. In the total sample, individuals with BMI ≥30 kg/m^2^ had a significantly higher risk of kidney stones (Model 3 aOR: 1.86; 95% CI: 1.48–2.34; *p* < 0.001) compared to those with BMI <25 kg/m^2^.

**Table 3:** Weighted logistic regression analysis of kidney stone risk across age groups and BMI categories.

	Model 1		Model 2		Model 3	
	aOR (95% CI)	*p*	aOR (95% CI)	*p*	aOR (95% CI)	*p*
All participants						
BMI < 25.0 kg/m^2^	Reference		Reference		Reference	
25.0 ≤ BMI<30.0 kg/m^2^	1.57 [1.23, 2.01]	<0.001	1.57 [1.23, 2.02]	<0.001	1.45 [1.12, 1.86]	0.004
BMI ≥ 30.0 kg/m^2^	2.17 [1.76, 2.18]	<0.001	2.27 [1.83, 2.82]	<0.001	1.86 [1.48, 2.34]	<0.001
Age 20–39						
BMI < 25.0 kg/m^2^	Reference		Reference		Reference	
25.0 ≤ BMI<30.0 kg/m^2^	1.69 [1.14, 2.51]	0.001	1.82 [1.23, 2.71]	0.004	1.76 [1.17, 2.63]	0.007
BMI ≥ 30.0 kg/m^2^	1.55 [1.07, 2.23]	0.002	1.70 [1.17, 2.46]	0.006	1.51 [1.02, 2.23]	0.04
Age 40–59						
BMI < 25.0 kg/m^2^	Reference		Reference		Reference	
25.0 ≤ BMI<30.0 kg/m^2^	1.30 [0.89, 1.91]	0.172	1.37 [0.92, 2.02]	0.116	1.27 [0.84, 1.92]	0.258
BMI ≥ 30.0 kg/m^2^	2.29 [1.61, 3.27]	<0.001	2.47 [1.72, 3.56]	<0.001	2.02 [1.36, 3.02]	<0.001
Age ≥ 60						
BMI < 25.0 kg/m^2^	Reference		Reference		Reference	
25.0 ≤ BMI<30.0 kg/m^2^	1.49 [1.02, 2.16]	0.039	1.33 [0.9, 1.96]	0.15	1.29 [0.87, 1.90]	0.202
BMI ≥ 30.0 kg/m^2^	2.01 [1.39, 2.89]	<0.001	1.94 [1.32, 2.85]	<0.001	1.73 [1.16, 2.57]	0.008

Adjusted covariates: model 1: univariate analysis; model 2: Sex, Race/Ethnicity, Education Level, INDFMPIR; model 3: model 2 plus Smoking History, Alcohol History, Coronary heart disease, High blood pressure, Moderate/Vigorous recreational activities. BMI, body mass index; CI, confidence interval; aOR, adjusted odds ratio.

In age-stratified analyses: Age 20–39 years: BMI ≥30 kg/m^2^ was associated with increased kidney stone risk (aOR: 1.51; 95% CI: 1.02–2.23; *p* = 0.04). Age 40–59 years: BMI ≥30 kg/m^2^ showed the strongest association (aOR: 2.02; 95% CI: 1.36–3.02; *p* < 0.001). Age ≥ 60 years: BMI ≥30 kg/m^2^ remained significantly associated with kidney stone risk (aOR: 1.73; 95% CI: 1.16–2.57; *p* = 0.008).

### 3.5 Interaction analysis between BMI categories and covariates

**Figure 3:**
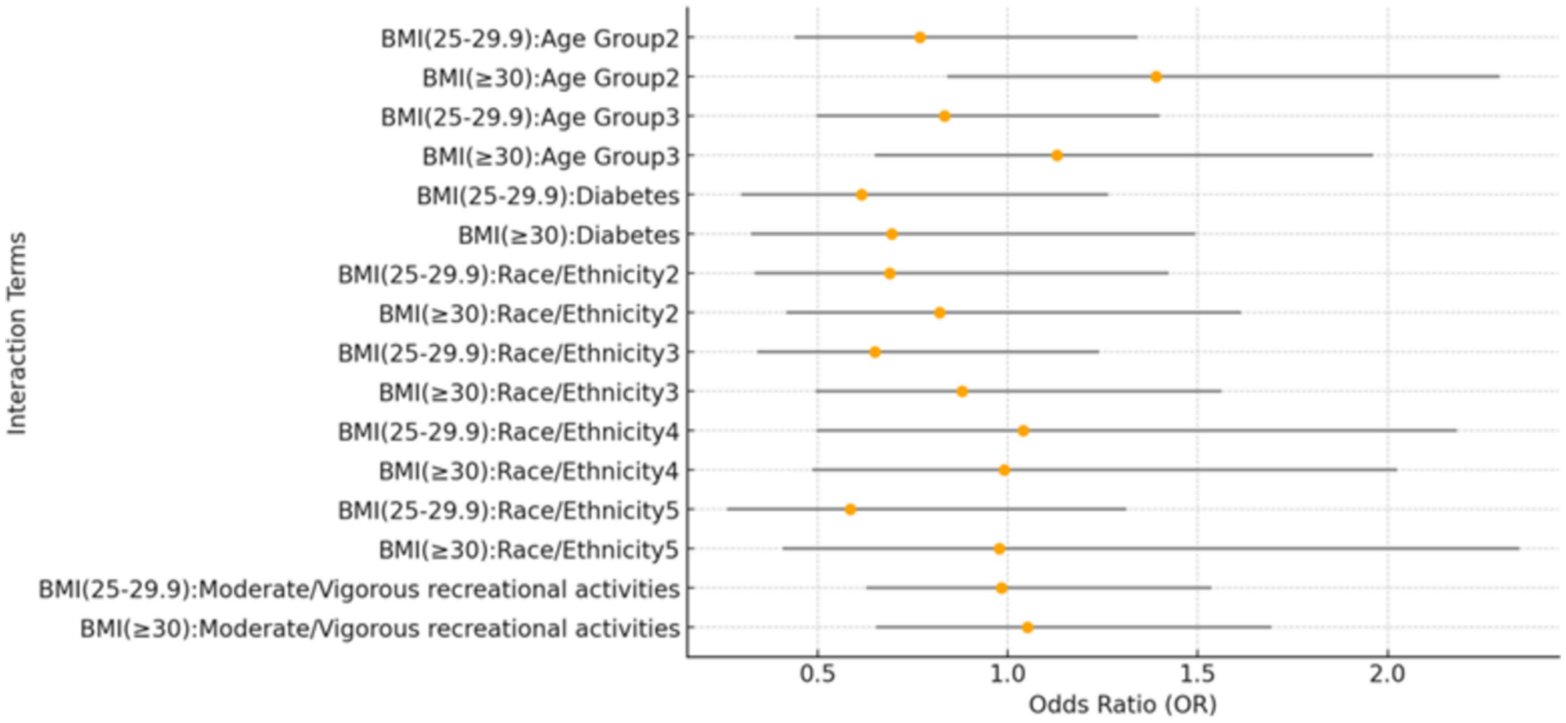
Forest plot of interaction effects between BMI categories and covariates. This forest plot presents the interaction effects of BMI categories with various covariates on the prevalence of kidney stones. The reference group for age is individuals aged 20–39 years, while the 40–59 years group and the ≥60 years group represent the two older age categories. For race/ethnicity, the reference group is Mexican American. The other categories are: Other Hispanic (2), Non-Hispanic White (3), Non-Hispanic Black (4), and Other Race – including multi-racial (5). The plot visualizes the estimates and confidence intervals for each interaction term.

Interaction analyses revealed no significant interactions between BMI categories and covariates such as age groups, race/ethnicity, physical activity, and DM, indicating the association between BMI and kidney stone risk is consistent across these subgroups, as shown in Figure 3.

### 3.6 Non-linear association between BMI and kidney stone risk across age groups

**Figure 4:**
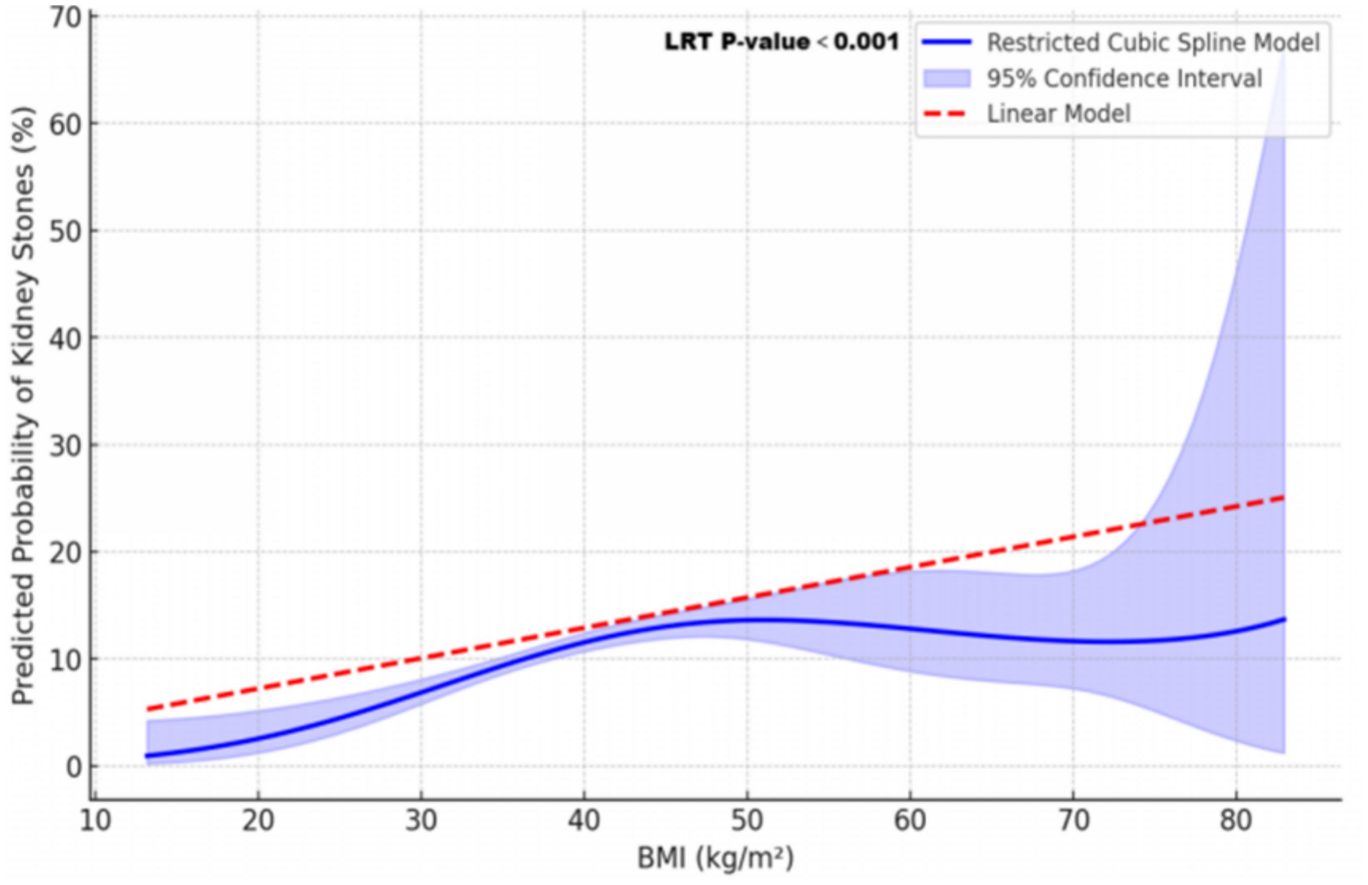
Non-linear and linear models of the relationship between BMI and predicted kidney stone risk. This figure illustrates the relationship between BMI and predicted kidney stone risk, modeled using a restricted cubic spline (RCS, solid blue line) and a linear model (red dashed line). The RCS model captures a non-linear association, with risk gradually increasing at lower BMI levels, plateauing around mid-range BMI, and fluctuating at higher BMI values. The shaded blue area represents the 95% confidence interval, which widens at extreme BMI levels due to greater variability. The superiority of the RCS model over the linear model is supported by a likelihood ratio test (LRT, *p*-value <0.001).

Figure 4 illustrates the association between BMI and kidney stone risk, comparing a non-linear restricted cubic spline (RCS) model (blue line) with a linear model (red dashed line). The RCS model showed a gradual increase in kidney stone risk at BMI levels below 50 kg/m^2^, followed by a fluctuating phase with wider confidence intervals beyond 50 kg/m^2^. A likelihood ratio test (LRT, *p*-value <0.001) confirmed the superior fit of the RCS model over the linear model.

**Figure 5:**
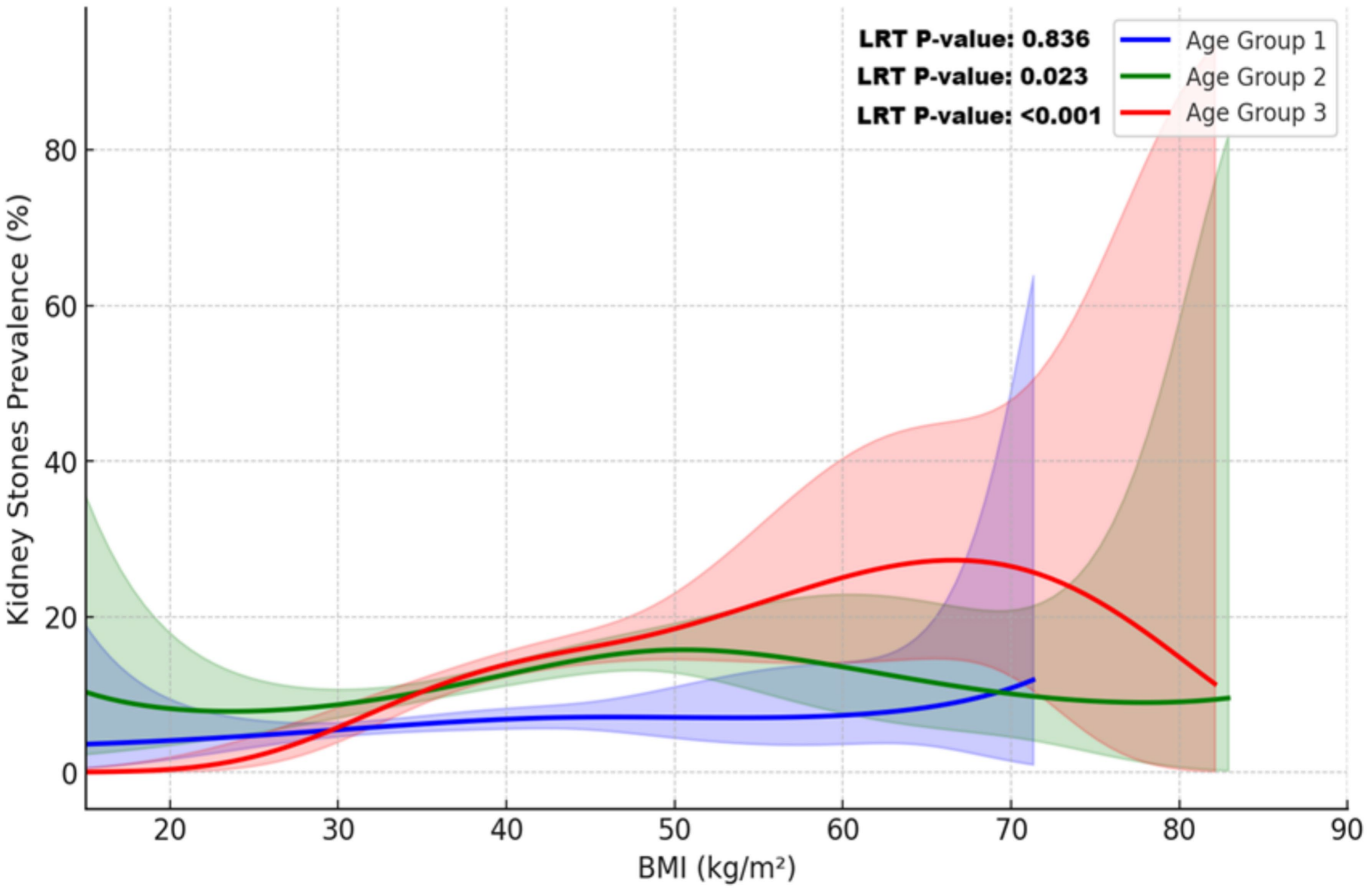
Non-linear relationship between BMI and kidney stone prevalence across age groups. This plot illustrates the relationship between BMI and kidney stone prevalence across age groups, modeled using restricted cubic splines (RCS) and adjusted for sampling weights. Covariates included sex, race/ethnicity, education, income, alcohol use, smoking, coronary heart disease, diabetes mellitus, hypertension, and physical activity. Solid lines represent the predicted probabilities for each age group: blue (20–39 years), green (40–59 years), and red (≥60 years). Shaded areas indicate 95% confidence intervals. The non-linear relationship was confirmed by a likelihood ratio test (LRT, *p*-value <0.001), with substantial variation observed at higher BMI levels, particularly in individuals aged ≥60 years.

Figure 5 highlights age-specific BMI-related risk patterns, revealing non-linear trends in older age groups and a linear trend in younger individuals. Among individuals aged ≥60 years, kidney stone risk exhibited a complex non-linear pattern, with significant variations across the BMI spectrum (LRT *p*-value <0.001). For the 40–59 years group, risk peaked at a BMI of approximately 45 kg/m^2^, demonstrating a statistically significant non-linear association (LRT *p*-value = 0.023). In contrast, the 20–39 years group showed a linear increase in risk, with no evidence of non-linearity (LRT *p*-value = 0.836).

### 3.7 Comparison of model fit across age groups

We compared the fit of logistic regression models (Models 1, 2, and 3) with a RCS model across three age groups using Akaike (AIC) and Bayesian (BIC) Information Criteria. Lower values indicate a better fit. For the overall sample, Model 3 provided the best fit among the logistic models, while the RCS model offered no significant improvement. In the 20–39 group, Model 3 had the best fit, but the RCS model slightly outperformed it in the 40–59 and ≥ 60 age groups. These results suggest that the RCS models performed better in the older age groups (Table 4).

**Table 4:** AIC and BIC comparison of logistic and RCS models across age groups.

	AIC	BIC
All patients		
Model 1	9888.889	9911.713
Model 2	9771.358	9862.652
Model 3	9620.411	9764.958
RCS	9624.087	9776.243
Age 20–39		
Model 1	2284.102	2303.533
Model 2	2253.728	2331.452
Model 3	2239.763	2362.826
RCS	2252.051	2381.591
Age 40–59		
Model 1	3519.655	3539.112
Model 2	3479.997	3557.823
Model 3	3459.695	3582.919
RCS	3459.407	2381.591
Age ≥ 60		
Model 1	4131.87	4151.558
Model 2	4047.079	4125.832
Model 3	4036.354	4161.046
RCS	4033.05	4164.305

AIC, Akaike Information Criterion; BIC, Bayesian Information Criterion; RCS, Restricted Cubic Spline.

Model 1: the basic logistic regression model with the fewest covariates.

Model 2: model 1 plus Sex, Race/Ethnicity, Education Level, INDFMPIR.

Model 3: model 2 plus Smoking History, Alcohol History, Coronary heart disease, High blood pressure, Moderate/Vigorous recreational activities.

## 4 Discussion

This study examined the association between BMI and kidney stone prevalence across different age groups using NHANES 2009–2018 data. A higher BMI was significantly associated with an increased risk of kidney stones, with individuals having a BMI ≥30 kg/m^2^ showing markedly elevated risk compared to those with a BMI <25 kg/m^2^. This association was evident across all age groups but was particularly pronounced in individuals aged 40 years and older.

These findings align with prior studies linking obesity to kidney stone formation through metabolic and physiological changes, such as hypercalciuria, hyperuricosuria, and disrupted urinary acid–base homeostasis (9, 10, 19). While systematic reviews confirm obesity as a key risk factor (20), most studies have overlooked age-specific variations. By conducting age-stratified analyses, our study reveals the nonlinear association between BMI and kidney stone risk, emphasizing age as a potential modifier and the need for tailored prevention strategies.

The relationship between elevated BMI and increased risk of kidney stones can be explained by multiple obesity-related physiological mechanisms. High BMI is frequently associated with metabolic syndrome, which encompasses insulin resistance, hypertension, and dyslipidemia (21, 22). These metabolic disturbances lead to significant alterations in urinary composition, including increased excretion of calcium, oxalate, and uric acid, along with decreased citrate excretion—all of which are established risk factors for kidney stone formation (21, 23). Additionally, obesity is associated with increased production of acidic metabolites and reduced ammonium excretion, leading to a lower urinary pH, which favors the formation of uric acid stones (24). Visceral adipose tissue in obese individuals secretes pro-inflammatory cytokines and adipokines, contributing to oxidative stress and impairing renal function (25, 26). This chronic inflammatory state can compromise renal tubular function, disrupting calcium and phosphate metabolism, thereby further facilitating stone formation. Moreover, dietary patterns commonly associated with high BMI, such as increased intake of animal protein and sodium, can exacerbate urinary metabolic disturbances, further heightening the risk of kidney stone development (16). These interrelated mechanisms underscore the complex role of obesity in promoting kidney stone formation.

The stronger association between obesity and kidney stone risk in middle-aged and older adults can be attributed to age-related physiological changes. Aging involves declining kidney function, hormonal shifts—such as reduced estrogen in postmenopausal women—and higher rates of comorbidities like hypertension and type 2 diabetes mellitus, all of which amplify obesity’s impact on stone formation. Hormonal changes can disrupt calcium metabolism and urinary excretion, increasing stone risk (13, 27). Prolonged exposure to metabolic risk factors further compounds the effect of high BMI in these age groups (28). In younger adults, better metabolic and kidney function may mitigate the direct effects of high BMI on stone risk. However, specific factors, such as the systemic immune-inflammation index (SII), have shown significant positive associations with kidney stone risk in individuals under 50 (29). Combined with rising obesity rates, these trends suggest an earlier onset of nephrolithiasis. Over time, prolonged high BMI may contribute to a gradual increase in risk, highlighting the need for early intervention to address obesity-related factors (30). These findings emphasize the multifactorial nature of nephrolithiasis and the importance of age-stratified analyses to unravel the complex interplay between age, obesity, and kidney stone risk.

Moreover, our analysis revealed a non-linear relationship between BMI and kidney stone risk, identified through the application of RCS modeling. The RCS model demonstrated that in individuals aged 40 years or older, the risk of kidney stones increased with BMI up to a certain point, after which it plateaued and even showed a slight decrease at extremely high BMI levels. This trend was especially evident in those aged 60 years or older. This nonlinear pattern underscores the complexity of the relationship between obesity and kidney stones, highlighting the limitations of assuming a simple linear association. A similar nonlinear association between BMI and other diseases has also been documented. In patients with advanced chronic kidney disease, higher BMI has been proposed to exert a protective effect, potentially associated with improved survival—a concept known as the “obesity paradox” (31). In patients with type 2 diabetes mellitus, a nonlinear relationship has been observed between BMI and all-cause mortality, with some studies suggesting a reverse J-shaped association between BMI and mortality risk (32, 33). These findings suggest that at extremely high BMI levels, the relationship between BMI and health outcomes may become more complex. Another consideration is the potential for selection bias. The small number of individuals with extreme obesity in studies may reduce statistical power, as reflected in wider confidence intervals. Additionally, the high mortality rate associated with severe obesity and related health conditions, such as cardiovascular disease, may reduce the number of older individuals with very high BMI, potentially influencing the observed association (34).

Our findings underscore the importance of BMI management in reducing kidney stone prevalence, particularly among individuals aged 40 years and older. Tailored prevention strategies should be developed to address specific age-related risk profiles. Middle-aged and older adults may benefit from comprehensive metabolic management, including control of blood glucose, blood pressure, and lipid levels (35, 36), while younger individuals would gain from early education on healthy lifestyles to reduce long-term kidney stone risk (17). Weight loss interventions, including dietary changes and increased physical activity, not only improve metabolic health and normalize urinary composition but also contribute to broader public health efforts aimed at reducing obesity and kidney stone prevalence (16, 37, 38).

The primary strength of this study lies in its use of a large, nationally representative NHANES sample, which enhances the generalizability of the findings. Advanced statistical methods, including weighted logistic regression and RCS modeling, allowed for a detailed evaluation of both linear and nonlinear associations, as well as BMI interactions with kidney stone risk across age groups. However, this study has several limitations. First, its cross-sectional design precludes causal inferences between BMI and kidney stone formation. While adjustments were made for multiple confounders, unmeasured factors—such as dietary habits, fluid intake, and genetic predisposition—may have introduced residual confounding. Second, reliance on self-reported kidney stone history may have led to recall or misclassification bias, potentially affecting prevalence estimates. Nonetheless, self-reported data have been widely validated and are commonly used in large-scale studies like NHANES (11, 17, 39). Third, while the study highlights nonlinear associations between BMI and kidney stone risk across different age groups, it did not explore dose–response relationships within each group. Future research should aim to identify specific BMI thresholds and incremental risk changes to inform more targeted clinical and public health strategies. Additionally, further studies investigating molecular and metabolic pathways could uncover therapeutic targets and guide personalized prevention strategies. To develop more comprehensive risk models, future research should also consider other potential contributors, such as dietary habits, genetic predisposition, fluid intake, and environmental exposures. Understanding how these factors, along with obesity-related metabolic changes, influence kidney function and urinary biochemistry at different life stages could improve risk stratification and guide multifaceted prevention approaches (16, 40, 41). This could ultimately lead to more effective interventions for reducing obesity-related kidney stone prevalence.

## 5 Conclusion

High BMI is significantly associated with an increased risk of kidney stones, particularly among middle-aged and older adults. The observed non-linear relationship, better captured by the restricted cubic spline model compared to the linear model, underscores the complexity of this association. These findings highlight the need for age-specific BMI management strategies to effectively reduce kidney stone risk.

## Data Availability

The datasets presented in this study can be found in online repositories. The names of the repository/repositories and accession number(s) can be found below: the dataset(s) supporting the conclusions of this article are available in NHANES repository at https://www.cdc.gov/nchs/nhanes/.

## Footnotes

^1^ https://www.cdc.gov/nchs/nhanes/
